# Draft Genome Sequences of *Escherichia coli* Strains Isolated from Septic Patients

**DOI:** 10.1128/genomeA.01278-14

**Published:** 2014-12-18

**Authors:** Madison I. Dunitz, David A. Coil, Guillaume Jospin, Jonathan A. Eisen, Jason Y. Adams

**Affiliations:** a University of California Davis Genome Center, Davis, California, USA; b University of California Davis, Department of Evolution and Ecology, Department of Medical Microbiology and Immunology, Davis, California, USA; c University of California Davis School of Medicine, Department of Internal Medicine, Division of Pulmonary, Critical Care, and Sleep Medicine, Sacramento, California, USA

## Abstract

We present the draft genome sequences of six strains of *Escherichia coli* isolated from blood cultures collected from patients with sepsis. The strains were collected from two patient sets, those with a high severity of illness, and those with a low severity of illness. Each genome was sequenced by both Illumina and PacBio for comparison.

## GENOME ANNOUNCEMENT

Sepsis is a clinical syndrome defined as infection complicated by a systemic inflammatory response syndrome. Sepsis affects >1.6 million Americans each year and is the most costly reason for hospitalization in the United States (1, 2). The more severe subtypes of sepsis, known as severe sepsis and septic shock, are associated with acute end-organ dysfunction and a 30% risk of in-hospital mortality (3, 4). Mortality increases with increasing severity of end-organ dysfunction at the population level (5, 6); however, the clinical outcomes of individual patients vary significantly. The relative contributions of host susceptibility factors and pathogen virulence-associated factors to the severity of illness in sepsis are not well understood. In this study, we sequenced 3 pairs of uropathogenic *Escherichia coli* strains isolated from blood samples from patients with sepsis from a urinary tract infection who were admitted to the UC Davis Medical Center. Each pair of strains was selected from patients who were matched with regard to clinical characteristics, including age group, sex, site of infection within the urinary tract, and major comorbidities, but they differed significantly in sepsis severity of illness, as defined by scores on the Sequential Organ Failure Assessment (SOFA) (7). *E. coli* strains JA23, JA62, and JA65 were from patients with a high severity of illness, whereas strains JA03, JA17, and JA69 were from patients with a low severity of illness. All aspects of the study were approved by the institutional review board of the UC Davis Medical Center (protocol no. 247849).

Strains of *E. coli* were isolated from overnight-plated subcultures of initial liquid blood cultures obtained in the course of routine clinical practice and stored in a biorepository at -80°C. A single colony from each strain was grown in LB broth at 37°C and then used for genomic DNA extraction.

Illumina paired-end libraries were made from *E. coli* genomic DNA extracted using a Promega Wizard genomic DNA purification kit (Promega Corporation, Madison, WI). The libraries were prepared using an Illumina TruSeq kit (Illumina, San Diego, CA). The samples were pooled and then sequenced on an Illumina MiSeq for paired-end 250-bp reads. An average of 2,355,125 paired-end reads per sample were generated. Quality trimming and error correction resulted in an average of 2,040,300 high-quality reads. All sequence processing and assembly of the Illumina reads were performed using the a5 assembly pipeline (8). Automated annotation was performed using the RAST server (9). The assembly and annotation statistics are presented in Table 1.

**Table 1:** Accession numbers and assembly statistics for 6 *E. coli* strains

Strain ID by sequencing method^*a*^	No. of contigs	No. of scaffolds	*N*_50_ contig (bp)	Total size (bp)	Coverage (×)	G+C content (%)	No. of ORFs	No. of RNAs	Accession no.	Version
Illumina sequencing										
JA03	134	123	183,087	5,287,643	105	51	5,257	107	JFFJ00000000	JFFJ00000000.1
JA17	99	94	288,084	5,064,765	101	51	4,946	100	JFFK00000000	JFFK00000000.1
JA23	62	42	372,835	4,933,283	28	51	4,774	98	JFFL00000000	JFFL00000000.1
JA62	139	120	201,894	5,336,948	60	51	5,414	111	JFFM00000000	JFFM00000000.1
JA65	84	83	172,163	4,802,037	95	51	4,666	107	JFFN00000000	JFFN00000000.1
JA69	102	97	198,017	5,203,662	59	51	5,132	106	JFFO00000000	JFFO00000000.1
Avg	103	93	236,013	5,104,723	75	51	5,032	105		

PacBio sequencing										
JA03_pb	10	NA^*b*^	3,249,757	5,411,202	67	51	5,387	117	JMIZ00000000	JMIZ00000000.1
JA17_pb	3	NA	5,119,992	5,119,992	86	51	4,959	109	JMJA00000000	JMJA00000000.1
JA23_pb	3	NA	4,904,536	4,904,536	55	51	4,775	111	JMJB00000000	JMJB00000000.1
JA62_pb	17	NA	5,559,237	5,559,237	52	51	5,869	110	JMJC00000000	JMJC00000000.1
JA65_pb	3	NA	4,876,096	4,876,096	68	51	4,728	113	JMJD00000000	JMJD00000000.1
JA69_pb	27	NA	5,319,871	5,319,871	47	51	5,368	148	JMJE00000000	JMJE00000000.1
Avg	11		4,838,248	5,198,489	63	51	5,181	118		

a ID, identification.

b NA, not available.

PacBio sample prep and genomic DNA extraction were performed using a Mo Bio PowerSoil DNA isolation kit (Mo Bio, Carlsbad, CA). PacBio libraries were prepared via the PacBio standard 10-kb library prep and sequenced using Pacific Biosciences RS sequencing technology (Pacific Biosciences, Menlo Park, CA). *De novo* assembly of the read sequences was performed using the Hierarchical Genome Assembly Process (HGAP2) workflow (PacBio DevNet; Pacific Biosciences) as available in single-molecular real-time (SMRT) Analysis. Automated annotation was performed using the RAST annotation server (9). The assembly and annotation statistics are presented in Table 1.

### Nucleotide sequence accession numbers.

All 12 assemblies described in this paper have been deposited as whole-genome shotgun projects in DDBJ/EMBL/GenBank under the accession numbers provided in Table 1.
